# Hydrazonoyl Chlorides as Precursors for Synthesis of Novel Bis-Pyrrole Derivatives

**DOI:** 10.3390/molecules21030326

**Published:** 2016-03-09

**Authors:** Nabila Abdelshafy Kheder

**Affiliations:** Department of Chemistry, Faculty of Science, Cairo University, Giza 12613, Egypt; nabila.abdelshafy@gmail.com; Tel.: +966-5422-33172

**Keywords:** bis-pyrrole, antibacterial activity, antifungal activity, hydrazonoyl halides, molecular docking

## Abstract

A convenient synthesis of some novel bis-pyrrole derivatives via hydrazonoyl halides is described. Antimicrobial evaluation of some selected examples of the synthesized products was carried out. The bis-pyrrole derivative having chloro substituents showed good activity against all of the used microbes. The molecular docking of the bis-pyrrole derivatives was performed by the Molecular Operating Environment (MOE) program.

## 1. Introduction

Bis-heterocycles have attracted much attention because of their diverse biological activities [1,2,3]. These include antibacterial, fungicidal, tuberculostatic, and plant growth regulative properties. Diverse pharmacological activities have been associated with pyrrole derivatives, such as anti-tubulin activity [4], non-Competitive mGluR1 antagonists [5], DNA-Binding applications [6], antitumor activity [7,8], antidepressant activity [9], treatment of hyperlipidemias [10], antiviral [11], and antimicrobial activity [12]. Hydrazonoyl chlorides are highly versatile and useful building blocks for the synthesis of a wide variety of heterocyclic compounds [13,14]. In view of the above-mentioned findings, and as a continuation of my interest in developing new routes for the synthesis of mono- and bis-heterocyclic systems for biological evaluation [15,16,17,18,19,20,21,22,23,24,25], I report herein a convenient route to some novel bis-pyrrole derivatives using hydrazonoyl chlorides and diethyl (*Z*,*Z*)-3,3′-(ethane-1,2-diyldiimino)dibut-2-enoate (**1**) [26,27].

## 2. Results and Discussion

### 2.1. Chemistry

**Scheme 1:**
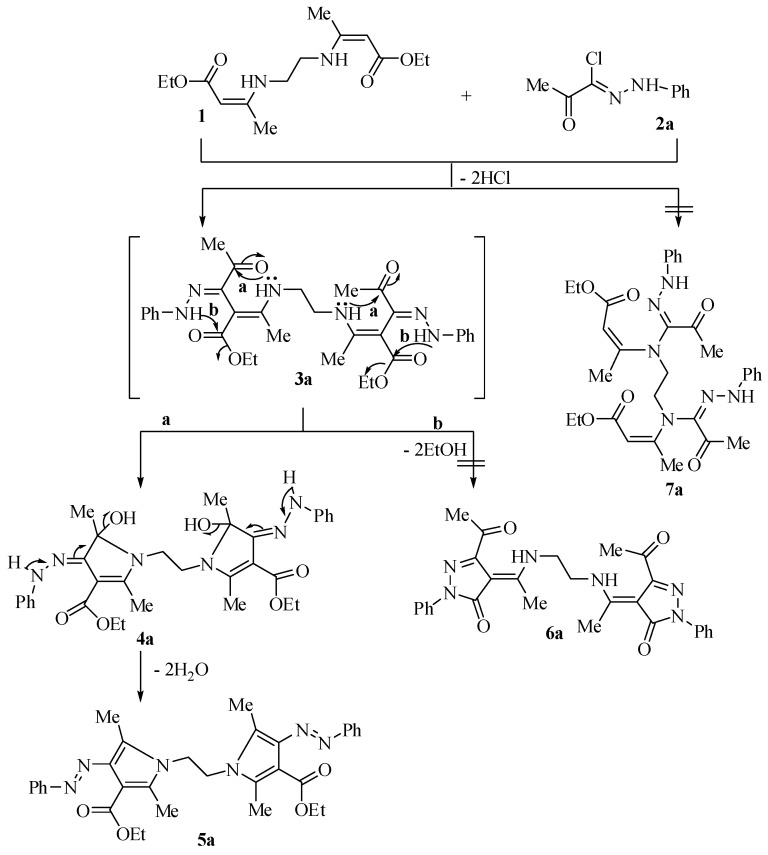
Synthesis of diethyl 1,1′-(ethane-1,2-diyl)bis(2,5-dimethyl-4-(phenylazo)-1*H*-pyrrole-3-carboxylate) (**5a**).

Treatment of compound **1** with a α-ketohydrazonoyl halide (**2a**) [28,29] in (1:2 molar ratios), in dry benzene at refluxing temperature in the presence of triethylamine (2 molar ratio) afforded the bis-pyrrole derivative **5a** (Scheme 1). The structure of compound **5a** was elucidated from its spectroscopic, as well as elemental analytical data. Its ^1^H-NMR spectrum revealed a triplet signal at δ 1.13 (*J* = 7.1 Hz) due to CH_3_ protons, two singlet signals at δ 2.17 and 2.34 due to two CH_3_ protons and a quartet signal at δ 4.14 (*J* = 7.1 Hz) due to CH_2_ protons, a singlet signal δ 4.31 due to NCH_2_ protons, in addition to an aromatic multiplet in the region 7.40–7.68 ppm. This spectroscopic analysis allows ruling out structures **6a** and **7a**. To account for the formation of the product **5a**, it is assumed that the reaction initially proceeds via an initial nucleophilic substitution to give the intermediate **3a**, which underwent intramolecular cyclization followed by the elimination of two water molecules to afford the final product **5a**.

**Scheme 2:**
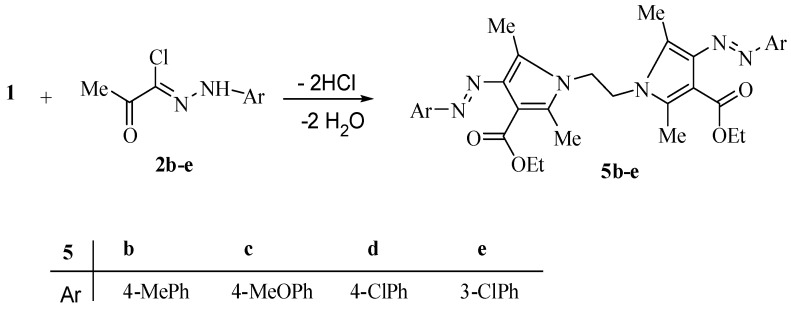
Synthesis of bis-pyrrole derivatives **5b**–**e**.

Prompted by the aforementioned results, and to generalize this reaction, the behavior of the hydrazonoyl halides **2b**–**e** [30] towards compound **1** was studied, under the same experimental conditions, which led to the respective bis-pyrrole derivatives **5b**–**e** (Scheme 2).

### 2.2. Antimicrobial Evaluation

Gram-positive and Gram-negative standard bacterial strains were used in this study to screen synthesized compounds for their potential antibacterial activities. The Gram-positive bacteria were *Staphylococcus aureus* and *Bacillus subtilis*. The Gram-negative bacteria were *Pseudomonas aeruginosa* and *Escherichia coli*. Four species of fungi known to cause different types of mycoses were also used to test the antifungal activities of synthesized compounds in this study. These fungal species were *Aspergillus fumigatus*, *Geotrichum candidum*, *Syncephalastrum racemosum*, and *Candida albicans*. Inhibition zone diameter (IZD) in mm was used a criterion for the antimicrobial activity using the agar diffusion well method. The fungicide Itraconazole and the bactericides Penicillin G and Streptomycin were used as references to evaluate the potency of the tested compounds under the same conditions. The results are depicted in Table 1.

The results revealed that most of the tested compounds revealed better activity against the Gram-positive bacteria rather than the Gram-negative bacteria. Also, all compounds exhibited almost no activity against *Candida albicans*. Compound **5d** was found to be the most potent relative to the standard drug, Itraconazole, against *Aspergillus fumigates*.

Additionally, compound **5d** has a high degree of antibacterial activity against Gram-positive bacteria *Staphylococcus aureus* (MSSA) and *Bacillus subtilis*. All the tested compounds except **5d** exhibited no activity against *Pseudomonas aeruginosa*. The structure antimicrobial activity relationship of the synthesized compounds revealed that the maximum activity was attained with compound **5d**, having chloro substituent in the para position of the phenyl ring.

### 2.3. Docking and Molecular Modeling

Molecular docking is used to predict the binding mode of ligands within the binding site of target proteins [31]. To validate and specify the target protein for the anti-bacterial activity of newly synthesized bis-pyrrole derivatives *E. coli* Enoyl reductase protein was selected and downloaded from the Protein Data Bank (PDB ID: 1LXC) [32].

**Figure 1:**
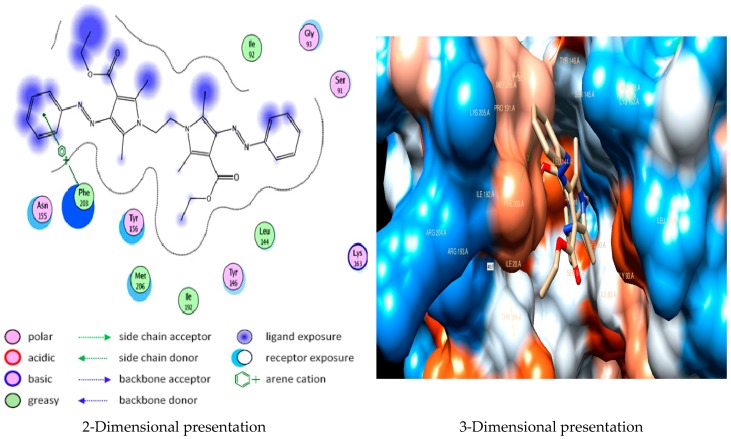
Docking of compound **5a** into *E. coli* Enoyl reductase Enzyme (PDB ID: 1LXC) showing arene-cation interaction between Phe203 and the phenyl ring.

**Figure 2:**
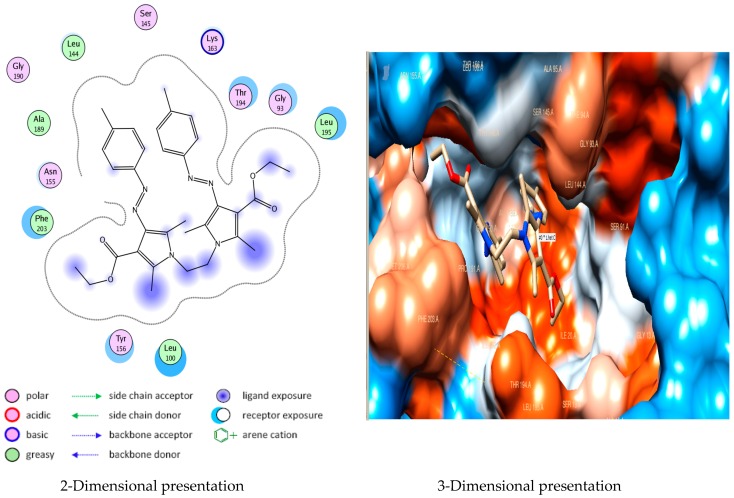
Docking of compound **5b** into *E. coli* Enoyl reductase Enzyme (PDB ID: 1LXC).

Docking studies of compound **5a** into the active site of *E. Coli* Enoyl reductase Enzyme showed van der Waals bonding between Phe203 and the phenyl ring (Figure 1), while docking studies of compound **5b** showed no interactions with *E. coli* Enoyl reductase Enzyme (Figure 2).

**Figure 3:**
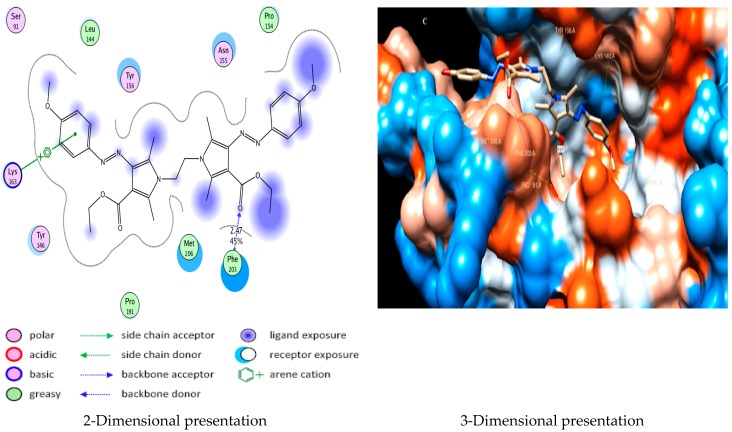
Docking of compound **5c** into *E. coli* Enoyl reductase Enzyme (PDB ID: 1LXC) showing arene-cation interaction with Lys163 and hydrogen bond with Phe 203.

Similarly, docking conformation of compound **5c** in the active site of *E. coli* Enoyl reductase Enzyme showed good interactions with the active site residues of this protein. Compound **5c** formed hydrogen bond interaction between carbonyl group moiety, as it acts as a hydrogen bond acceptor with the side chain of Phe203 residue (2.47 Å) with a strength of 45%. Furthermore, it showed van der Waals interaction with Lys163 (Figure 3).

**Figure 4:**
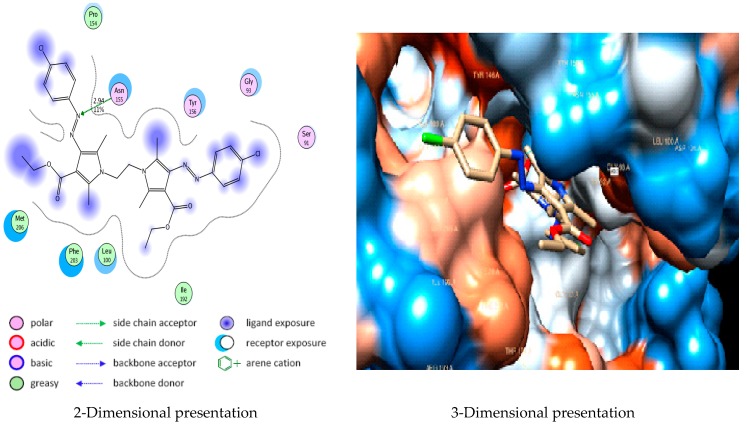
Docking of compound **5d** into *E. coli* Enoyl reductase Enzyme (PDB ID: 1LXC) showing hydrogen bond with Asn155.

**Figure 5:**
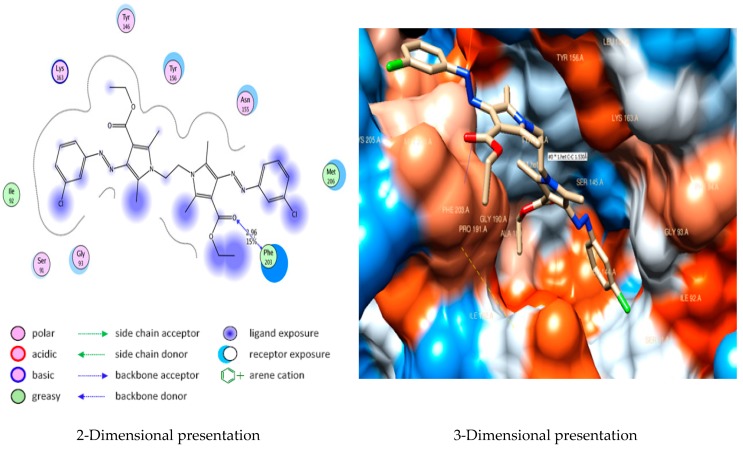
Docking of compound **5e** into *E. coli* Enoyl reductase Enzyme (PDB ID: 1LXC) showing hydrogen bond with Phe203.

Docking studies of compound 5d showing hydrogen bond interaction between nitrogen atom of azo group moiety, as it acts as a hydrogen bond acceptor with the side chain of Asn155 (2.49 Å) with a strength of 11% (Figure 4). while docking studies of compound 5e showing hydrogen bond interaction between carbonyl group moiety, as it acts as a hydrogen bond acceptor with the side chain of Phe203 (2.96 Å) with a strength of 15% (Figure 5).

## 3. Materials and Methods

### 3.1. Chemistry

#### 3.1.1. General

Melting points were measured on a Gallenkamp melting point apparatus and are uncorrected. IR spectra were recorded in potassium bromide discs on Shimadzu FTIR 8101 PC infrared spectrophotometer (Shimadzu, Tokyo, Japan). The NMR spectra were recorded on a BRUKER VX-500 NMR spectrometer (Varian, Palo Alto, CA, USA). ^1^H spectra were run at 500 MHz and ^13^C spectra were run at 125 MHz in deuterated dimethylsulphoxide (DMSO-*d*_6_) using TMS as an internal standard. Chemical shifts were related to that of the solvent. Mass spectra were recorded on a Shimadzu GCMS-QP 1000 EX mass spectrometer (Shimadzu, Tokyo, Japan) at 70 e.V. Elemental analyzes were measured by using a German made Elementar vario LIII CHNS analyzer (GmbH & Co. KG, Hanau, Germany). The biological evaluation of the products was carried out in the Medical Mycology Laboratory of the Regional Center for Mycology and Biotechnology of Al-Azhar University, Cairo, Egypt. Diethyl (*Z*,*Z*)-3,3′-(ethane-1,2-diyldiimino)-dibut-2-enoate (**1**) [26,27], hydrazonoyl chlorides (**2a**) [28,29], and **2b**–**e** [30] were prepared according to the procedures reported literature.

#### 3.1.2. Synthesis of Bis-Pyrrole Derivatives **5a**–**e**

General procedure: To a solution of compound **1** (0.284 g, 1 mmol) and the appropriate hydrazonoyl halide **2a**–**e** (2 mmol) in dry benzene (20 mL), was added triethylamine (0.2 mL, 2 mmol) and the reaction mixture was heated under reflux for 6 h. After cooling, the mixture was filtered off to remove the precipitated triethylamine hydrochloride and the solvent was distilled under reduced pressure. The oil residue was triturated with MeOH and the solid product was collected by filtration, washed with methanol, and recrystallized for purification to afford the corresponding bis-pyrrole derivatives **5a**–**e**.

*Diethyl 1,1*′*-(ethane-1,2-diyl)bis(2,5-dimethyl-4-(phenylazo)-1H-pyrrole-3-carboxylate)* (**5a**). Yield (40%), mp. 230–232 °C (EtOH/DMF); IR (KBr) ν_max_: 2960 (aliphatic CH), 1695 (C=O) cm^−1^; ^1^H-NMR (DMSO-*d*_6_): δ 1.13 (t, 6H, 2CH_3_, *J* = 7.1 Hz), 2.17 (s, 6H, 2CH_3_), 2.34 (s, 6H, 2CH_3_), 4.14 (q, 4H, 2CH_2_, *J* = 7.1 Hz), 4.31 (s, 4H, 2CH_2_), 7.40–7.68 (m, 10H, ArH); ^13^C-NMR (DMSO-*d*_6_): δ 8.97, 9.56, 14.13, 42.94, 59.62, 106.04, 121.48, 129.12, 129.41, 131.99, 133.27, 136.56, 152.45, 165.73; MS *m/z* (%) 570 (8.77), 569 (M^+^, 9.89), 463 (4.98), 105 (13.22), 77 (90.33). Anal. Calcd for C_32_H_36_N_6_O_4_: C, 67.59; H, 6.38; N, 14.78. Found: C, 67.64; H, 6.46; N, 14.66%.

*Diethyl 1,1*′*-(ethane-1,2-diyl)bis(2,5-dimethyl-4-(4-methylphenylazo)-1H-pyrrole-3-carboxylate)* (**5b**). Yield (38%), mp. 230–231 °C (EtOH/DMF); IR (KBr) ν_max_: 2978 (aliphatic CH), 1693 (C=O) cm^−1^; ^1^H-NMR (DMSO-*d*_6_): δ 1.13 (t, 6H, 2CH_3_, *J* = 6.9 Hz), 2.15 (s, 6H, 2CH_3_), 2.31 (s, 6H, 2CH_3_), 2.35 (s, 6H, 2CH_3_), 4.11 (q, 4H, 2CH_2_, *J* = 6.9 Hz), 4.28 (s, 4H, 2CH_2_), 7.26 (d, 4H, *J* = 7.8 Hz), 7.55 (d, 4H, *J* = 7.8 Hz); MS *m/z* (%) 598 (M^+^ + 1, 100), 100), 505 (13.52), 477 (6.3), 297 (2.3), 119 (30.76), 91 (56.18). Anal. Calcd for C_34_H_40_N_6_O_4_: C, 68.43; H, 6.76; N, 14.08. Found: C, 68.37; H, 6.68; N, 14.18%.

*Diethyl 1,1*′*-(ethane-1,2-diyl)bis(2,5-dimethyl-4-(4-methoxyphenylazo)-1H-pyrrole-3-carboxylate)* (**5c**). Yield (35%), mp. 228–230 °C (EtOH/DMF); IR (KBr) ν_max_: 2921(aliphatic CH), 1696 (C=O), 1600 (C=N) cm^−1^; ^1^H-NMR (DMSO-*d*_6_): δ 1.13 (t, 6H, 2CH_3_, *J* = 6.6 Hz), 2.15 (s, 6H, 2CH_3_), 2.30 (s, 6H, 2CH_3_), 3.8 (s, 6H, 2CH_3_), 4.12 (q, 4H, 2CH_2_, *J* = 6.6 Hz), 4.27 (s, 4H, 2CH_2_), 7.02 (d, 4H, *J* = 8.1 Hz), 7.56 (d, 4H, *J* = 8.1 Hz); ^13^C-NMR (DMSO*-d*_6_): 8.92, 9.53, 14.13, 42.93, 55.39, 59.48, 106.03, 114.24, 123.07, 130.30, 132.89, 136.44, 146.64, 160.35, 165.80. Anal. Calcd for C_34_H_40_N_6_O_6_: , C, 64.95; H, 6.41; N, 13.37. Found: C, 64.82; H, 6.38; N, 13.35%

*Diethyl 1,1*′*-(ethane-1,2-diyl)bis(2,5-dimethyl-4-(4-chlorophenylazo)-1H-pyrrole-3- carboxylate)* (**5d**). Yield (43%), mp. 214–215 °C (EtOH/DMF); IR (KBr) ν_max_: 2978 (aliphatic CH), 1701 (C=O) cm^−1^; ^1^H-NMR (DMSO-*d*_6_): δ 1.12 (t, 6H, 2CH_3_, *J* = 6.9 Hz), 2.16 (s, 6H, 2CH_3_), 2.32 (s, 6H, 2CH_3_), 4.12 (q, 4H, 2CH_2_, *J* = 6.9 Hz), 4.31 (s, 4H, 2CH_2_), 7.52 (d, 4H, *J* = 8.7 Hz), 7.68 (d, 4H, *J* = 8.7 Hz); ^13^C-NMR (DMSO-*d*_6_): δ 8.97, 9.55, 14.12, 42.95, 59.63, 106.06, 120.64, 123.04, 129.16, 132.72, 133.49, 136.45, 151.07, 165.55; MS *m/z* (%) 637 (M^+^, 5.46), 622 (0.89), 497 (0.52), 139 (2.18), 111 (8.72). Anal. Calcd for C_32_H_34_Cl_2_N_6_O_4_: C, 60.28; H, 5.38; N, 13.18. Found: C, 60.25; H, 5.29; N, 13.07%.

*Diethyl 1,1′-(ethane-1,2-diyl)bis(2,5-dimethyl-4-(3-chlorophenylazo)-1H-pyrrole-3-carboxylate)* (**5e**). Yield (44%), mp. 198–199 °C (EtOH); IR (KBr) ν_max_; 2974 (aliphatic CH), 1709 (C=O) cm^−1^; ^1^H-NMR (DMSO-*d*_6_): δ 1.17 (t, 6H, 2CH_3_, *J* = 6.9 Hz), 2.19 (s, 6H, 2CH_3_), 2.36 (s, 6H, 2CH_3_), 4.17 (q, 4H, 2CH_2_, *J* = 6.9 Hz), 4.34 (s, 4H, 2CH_2_), 7.15-7.66 (m, 8H, ArH); ^13^C-NMR (DMSO-*d*_6_): δ 9.12, 9.52, 14.12, 42.94, 59.71, 106.20, 119.57, 121.70, 128.88, 130.92, 133.06, 133.88, 133.92, 136.41, 153.61, 165.52. Anal. Calcd for C_32_H_34_Cl_2_N_6_O_4_: C, 60.28; H, 5.38; N, 13.18. Found: C, 60.17; H, 5.24; N, 13.25%.

### 3.2. Agar Diffusion Well Method to Determine the Antimicrobial Activity

The microorganism inoculums were uniformly spread using sterile cotton swabs on a sterile Petri dish malt extract agar (for fungi) and nutrient agar (for bacteria). One hundred µL of each sample was added to each well (10 mm diameter holes cut in the agar gel, 20 mm apart from one another). Then, the systems were incubated for 24–48 h at 37 °C (for bacteria) and at 28 °C (for fungi). After incubation, the microorganism’s growth was observed. Inhibition of the bacterial and fungal growth were measured in mm. Tests were performed in triplicate [33].

### 3.3. Docking Studies

Docking studies for the synthesized products were performed using Molecular Operating Environment (MOE) version 2008.10 (Chemical Computing Group Inc., Montreal, QC, Canada). Compounds **5a**–**e** were built using the builder interface of the MOE program and subjected to energy minimization using the included Forcefield MMFF94x calculations. The X-crystallographic structure of *E. coli* Enoyl reductase (PDB ID: 1LXC) which is complexed with ((*E*)-3-(6-aminopyridin-3-yl)-*N*-methyl-*N*-((1-methyl-1H-indol-2-yl)methyl) acrylamide (PDB ID: AYM)) (NAD), that was obtained from Protein Data Bank.

## 4. Conclusions

A new simple approach to bis-pyrrole derivatives from hydrazonoyl halides has been achieved. The maximum antimicrobial activity was attained with compound **5d**, having chlorine in the para position. The molecular docking of bis-pyrrole derivatives **5a**–**e** was performed using the MOE program.

## Figures and Tables

**Table 1:** Antibacterial and Antifungal Activities of the Synthesized Compounds.

Microorganisms	Compound Tested				Standard (30 µg/mL)
	5a	5b	5d	5e	
Fungi					Itraconazole
*Aspergillus fumigatus* (RCMB 002003)	18.2 ± 0.84	11.3 ± 0.68	21.1 ± 0.2	16.3 ± 0.09	22 ± 0.1
*Geotrichumcandidum* (RCMB 052006)	18.9 ±0.35	12.6 ±0.54	19.3 ± 0.05	15.4 ± 0.1	26 ± 0.3
*Syncephalastrum racemosum* (RCMB 005003)	11.2 ± 0.44	NA	16.4 ± 0.08	14.2 ± 0.05	19 ± 0.1
*Candida albicans* (RCMB 005002)	NA	NA	NA	NA	24 ± 0.1
Gram-positive Bacteria					Penicillin G
*Staphylococcus aureus* (RCMB 000106) (MSSA)	17.8 ± 0.58	10.7 ± 0.36	21.4 ± .03	17.9 ± 0.03	27.4 ± 0.08
*Bacillis subtilis* (RCMB 000107)	17.9 ± 0.46	11.1 ± 0.72	26.1 ± 0.04	15.2 ± 0.04	28.6 ± 0.03
Gram-negative Bacteria					Streptomycin
*Pseudomonas aeruginosa* (RCMB 000102)	NA	NA	19.9 ± 0.09	NA	26.3 ± 0.03
*Escherichia coli* (RCMB 000103)	12.6 ± 0.57	12.4 ±0.04	17.2 ± 0.2	13.4 ± 0.04	30.1 ± 0.07

NA: No activity, data are expressed in the form of mean ± standard deviation (S.D.)
